# A Field Study Examining the Attraction of Adult *Dermacentor variabilis* to Heat Stimuli Associated with Road Edge Habitats

**DOI:** 10.3390/pathogens14111147

**Published:** 2025-11-12

**Authors:** Noah L. Stewart, Hannah Stahlman, Richard L. Stewart, Marcie L. Lehman, Alison Luce-Fedrow

**Affiliations:** 1 Department of Biology, Shippensburg University of Pennsylvania, 1871 Old Main Drive, Shippensburg, PA 17257, USA; nstewart@pennstatehealth.psu.edu (N.L.S.); hannah.stewart@alumni.pitt.edu (H.S.); rlstew@ship.edu (R.L.S.); mllehm@ship.edu (M.L.L.); 2 Penn State College of Medicine, 500 University Drive, Hershey, PA 17033, USA

**Keywords:** American Dog Tick, roadside ecology, *Dermacentor variabilis*, Haller’s Organ, Rickettsia, tick control

## Abstract

Ticks use multiple sensory organs to facilitate host detection, including Haller’s organs (HOs) that allow ticks to sense infrared (IR) radiation from potential hosts. Additionally, ticks have primitive eyes to sense light sources. The possibility exists that these senses may detect stimuli that attract ticks to road edge habitat, where IR radiation tends to be elevated. We investigated the role of the HOs and eyes in the attraction of adult American dog ticks, *Dermacentor variabilis*, towards road edge habitat(s). Adult *D. variabilis* were collected from multiple study sites and separated into three groups: (1) Haller’s organs removed; (2) eyes painted with black nail polish; and (3) unmodified ticks (control). All tick groups were marked with a unique fluorescent paint color and released 7.5 m from the road edge at two study sites. Tick movements were tracked at night using ultraviolet lights, tick position(s) were recorded using flags, and measurements were recorded to track tick movement in relation to the release point and road edge. Surface temperatures were recorded at the road edge and in the field to detect a potential thermal stimulus. Mixed-effects models were applied to investigate the significance of tick proximity to the road edge between the groups and sites. Our results demonstrated that the control unmodified group was significantly closer to the road edge than the modified groups lacking Haller’s organ or eyes (*p* ≤ 0.0001, *p* = 0.0049), leading to the conclusion that unmodified ticks move towards road edges. Modifying ticks, either by removing the HO or eyes of adult *D. variabilis* decreased tick movement toward road edges.

## 1. Introduction

Hard ticks like *Dermacentor variabilis* (American dog tick), possess multiple sensory organs for monitoring internal and external environments. These organs include sensory neurons that innervate areas such as the palps, eyes, pharynx, and chelicerae [1]. All sensory nerves ultimately connect to the synganglion, a collection of nerves that acts as a “brain” for the tick [1]. The combination of these organs facilitates the ability of the tick to sense and respond to the environment. Two highly specialized tick sensory organs, potentially important to sensing hosts, are the Haller’s Organ (HO) and the eyes. The HO, also known as the foretarsal sensory organ, is an olfactory organ that is unique to Acari and is present in every post-embryonic stage of all tick species [1,2,3]. Haller’s organs are located on the tarsi of leg one and can sense multiple stimuli, including odorants, CO_2_, infrared (IR) radiation, and heat.

*Dermacentor variablis* adults have simple eyes that can detect light [1]. Eyes are located dorso-laterally above the second pair of legs [2]. While it is unknown what wavelengths of light can be detected by *D. variabilis* eyes, it does not appear to be important for host seeking as demonstrated by observations that adult *D. variabilis* can detect humans from several meters away, and that detection originates in the HO [4]. Moreover, Mitchell et al. [2] demonstrated that *D. variabilis* exhibited phototaxis, via the HO and not the eyes, to 850 nm infrared (IR) light in a choice bioassay. In an environment containing roads, black asphalt pavements strongly absorb solar radiation, leading to the accumulation of heat and high pavement surface temperatures [5]. Consequently, it is possible that high levels of retained solar energy could act as an IR attractant for *D. variabilis*, potentially detected through the HO. The function of eyes in *D. variabilis* may be for photoperiodism possibly congruent with what is reported for the two-spotted spider mite *Tetranychus urticae* [6].

Ticks congregate along forest and meadow edge habitat, where hosts are likely to gather [7]. Higher densities of *D. variabilis* and *A. americanum* have been reported in edge habitat along forest, young woodland, and meadow habitat in comparison to undisturbed habitat [8], potentially due to higher larval survival and/or the attraction of hosts to those habitats. Additionally, there is an increased likelihood of host presence in edge habitat, which also fosters the possibility of engorged female ticks dropping from their hosts and laying eggs in edge habitats [9]. High immature tick (*I. pacificus* and *D. occidentalis*) host loads have been observed on lizards in road edge microhabitats when compared to meadow edge, riparian, and structure edge microhabitats, but is likely a result of lizards spending more time along road edges than other habitats [10]. Similarly, an increased tick presence along road edges could be due to the abundance of domestic hosts (like dogs) [11], the production of CO_2_ from automobile traffic [12], and/or IR radiation generated from road heat [13]. Detection of IR radiation and CO_2_ via the HO allows ticks to detect the presence of hosts, which may also play a role in tick abundance at road surfaces. Past studies have documented the movement of ticks towards road edge habitat through marking, releasing, and dragging for ticks within the release site [12,13]. These studies demonstrated the attraction of marked but otherwise unaltered adult *D. variabilis*; they did not investigate the role of these sensory organs in the attraction of *D. variabilis* to road edge habitat. In this study, we examined movement of *D. variabilis* towards or away from road edges without functional eyes, without HO, or with no sensory organ modifications (control). We hypothesized that adult *D. variabilis* would move towards and congregate along road edge habitat primarily via stimulus detected by means of the HO.

## 2. Materials and Methods

**Figure 1:**
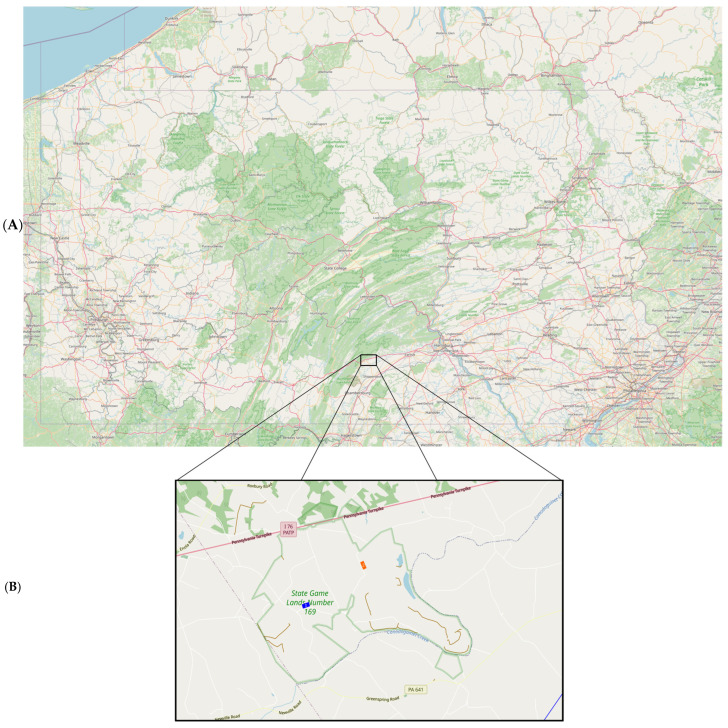
Study sites in south central Pennsylvania. (**A**) The location of SGL 169 to entire state of Pennsylvania. (**B**) Magnified view of study sites 1 (blue) and 2 (orange). Map data copyrighted OpenStreetMap contributors and available from https://www.openstreetmap.org (Accessed 4 November 2025) [14].

Overall Experimental Design. To test our hypothesis and evaluate the findings of Mitchell et al. 2017 [2] in a field setting, adult *D. variabilis* were collected from areas near our study sites. *Dermacentor variabilis* adults were modified as described by Mitchell et al. 2017 [2]. Modifications include surgical removal of the first tarsus on the front pair of legs to eliminate the HO sensory input and painting the eyes to eliminate any light detection. All released ticks were marked with fluorescent paint for tracking in the field and released 7.5 m from the road edge. At determined dates post release, tick positions were marked with flags to triangulate their current position. Positions, in relation to release point and road edge, were transferred as cartesian coordinates, thereby permitting measurement of tick movement over time. Temperature and CO_2_ data was collected at predetermined locations moving away from the road edge to evaluate for a potential stimulus. Detailed methods for each part of the experiment are as follows:

Study areas. The study area included two separate sites located within State Game Lands Number 169 (SGL 169) (40.1661985° N, −77.4874864° W), Newville, Pennsylvania (Figure 1). This game lands consists of approximately 2500 acres; study sites were selected based on locations along a paved road with similar estimated automobile traffic, known populations of *D. variabilis*, straight segments of road with >100 m in length, and distance from each site to be considered separate subpopulations. Each study site included a 100 m section along a black asphalt road traveled by light automobile traffic (defined by less than 10 cars per hour during daytime sampling events) and extended to 30 m back from the road edge. Each location consisted primarily of grassland habitat, defined by the presence of a mixture of grasses and primarily perennial herbaceous vegetation.

Tick Collection and Processing. Ticks used in the field tracking experiments were collected prior to placement in the field by dragging road edge habitat at SGL 169 near the two study sites. A one meter square of cloth was dragged over vegetation, which allowed for questing ticks to attach and be collected [15]. Dragging continued at areas near the field tracking sites until at least 150 *D. variabilis* adults were collected (which was typically completed by two collectors in several hours). Collected *D. variabilis* adults were stored in humidified Ziploc^TM^ bags in the field/laboratory under ambient temperatures in shade conditions, without direct sunlight. Adult *D. variabilis* released at the study sites were collected approximately 48 h prior to release and processed the same day in the laboratory. Ticks were placed into three categories: (1) ticks with eyes covered by two layers of black enamel nail polish, (2) ticks with the HO surgically removed, and (3) a control group with no sensory modifications as described by Mitchell et al. 2017 [2]. The methodology of applying nail polish to tick eyes was validated by Mitchell et al. 2017 [2]. Tick processing (paint or HO removal) and fluorescent paint marking occurred approximately 24 h prior to release. Haller’s organs were removed using an X-Acto No. 1 precision knife (X-Acto, Westerville, OH, USA) by excising the first tarsus from both front legs. Surgery was facilitated by placing the tick within a Petri dish under a dissection microscope. Surgical removal of the first tarsus of the front legs had no significance on activity or lifespan of adult ticks as none was detected in this study or by others as reported by Romenenko et al. [16] and Mitchell et al. [2]. Altered behaviors attributed to the removal of a structure are likely due to the absence of a sensory structure. Ticks were stored overnight in humidified Ziploc^TM^ bags to facilitate rehydration prior to experimental release and were monitored the next morning to ensure that there was no noticeable impairment of movement. All ticks were also marked with fluorescent Testors^TM^ enamel paint (Testors Co., Rockford, IL, USA) that was easily identified with a 365 nm UV light (DARKBEAM (TIANYIDA Technology development Co., Ltd., Shenzhen, China). Each tick group was marked with a unique color, and color was randomly assigned for each site to prevent sampling bias. Ticks were painted by holding the tick in soft forceps from the anterior end near the basis capitulum, so that the scutum and alloscutum could be painted with enamel paint. A uniform drop of paint was applied to the dorsal side of the tick using a toothpick. Painted ticks were then placed in a “tick arena” consisting of an insect collecting tray with adhesive edges until the paint dried.

**Figure 2:**
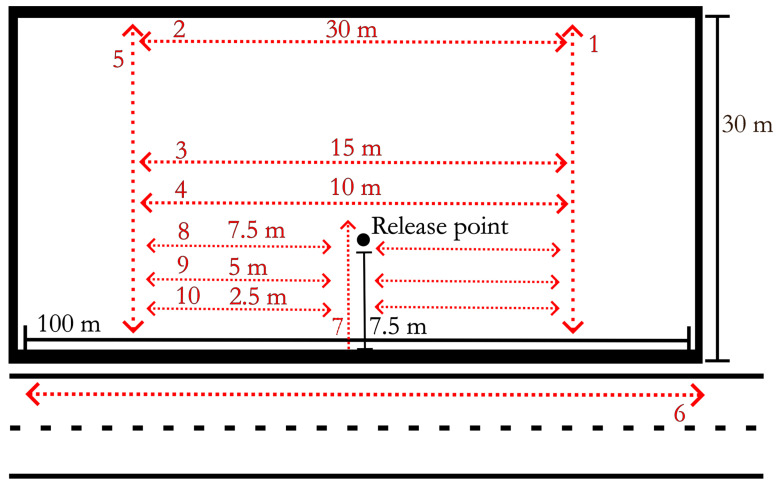
Example diagram of a typical field search pattern with transects numbered in the standard order of completion. Environmental data was collected along the 25 m and 75 m transects (1 and 5). Scanning was completed along transects parallel to the road at 2.5 m (10), 5 m (9), 7.5 m (8), 10 m (4), 15 m (3), and 30 m (2) from the road edge. Researchers also scanned within 2 m of the road edge by walking along the road (6).

Tick Tracking. Ticks were released 7.5 m from the road edge and 50 m along the 100 m site. Ticks were released on 27 May and 8 June at sites 1 and 2, respectively. Ticks were released on a wooden plank measuring twenty centimeters by forty centimeters. Fifty ticks from each experimental group were initially marked for release. Any ticks that were still present on the board during the first flagging night were excluded from the study. Tick positions were observed/recorded twice a week during the nighttime search events with 365 nanometer flashlights (DARKBEAM (TIANYIDA Technology development Co., Ltd.), Shenzhen, Guangdong, China) used to visualize the fluorescent paint label on the dorsum. Nighttime searches were conducted from the tick release date until 8 August 2021, when no ticks were found at either site during nighttime search events. Searches were conducted on fixed transects (Figure 2) by two researchers and consisted of moving along transects parallel to the road. 365 nanometer flashlights with new batteries for each sampling event were used to detect fluorescent marks on ticks. When a marked tick was located, the date and tick grouping (as identified by the fluorescent paint color) were recorded on white construction flags (Swanson Tool Co., Frankfort, IL, USA), placed in the ground to mark the location, and recorded in a field notebook.

**Figure 3:**
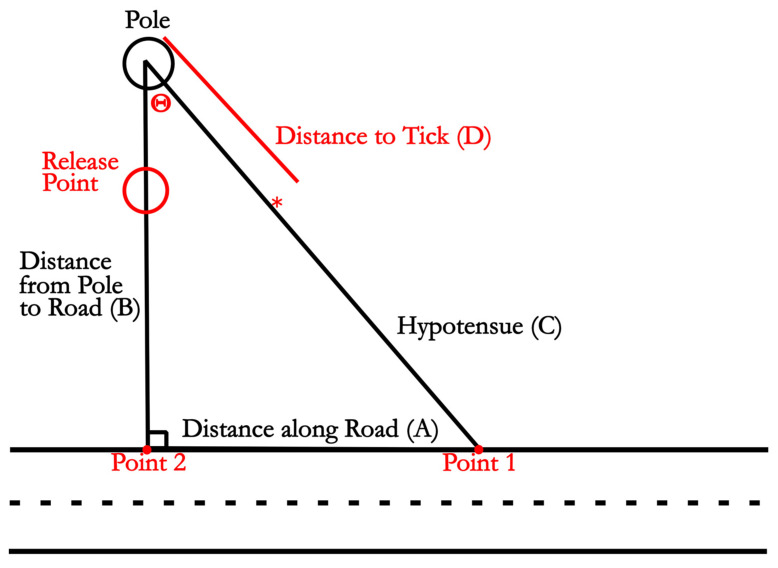
A representation of how measurements recorded in the field were used to convert the three length measurements into angle θ using θ = arccos (C2 + B2 − A2)/(2*C*B) with ∗ representing a flagged tick. X and Y components of the Cartesian coordinates were calculated by taking the distance from the pole to the tick and multiplying by sin(θ) and cos(θ) for the x and y coordinates, respectively.

Flag Collection and Information Processing. Flags were placed at identified tick sites twice each week and collected at the end of each week during daylight hours. Flag position in relation to the release point and road was recorded using two 100 m tape measures. One tape measure was laid along the road surface, and the other was attached to a wooden stake 2.5 m directly behind the tick release site, which was placed in this way to allow for measurement of ticks around and behind the release site. The tape measure attached to the stake was suspended at the location of the flag, and a plumb bob (Pittsburgh, Harbor Freight Tools, Calabasas, CA, USA) was used to hold the tape directly above the flag. The location of the flag along the tape measure, as well as where the two tape measures crossed, was recorded and used to triangulate the location of the tick. The location of the near and far edges of the road was also recorded at multiple points using the same method (Figure 3).

An unknown event of vandalism resulted in the removal of flags at study site 1 sometime between 11 p.m. on 25 June 2021, and 3:30 p.m. on 27 June 2021. All flags placed on 25 June 2021 were missing and significant vegetation trampling was observed around the release site (where many flags had been placed). Tick positions were reflagged on 27 June 2021, and their positions were recorded the following day. This event is noteworthy due to the removal of data from our study, as well as the possible interference with tick position and removal of ticks on the vandal’s clothing during flag removal.

Temperature Recordings. Surface temperature was recorded during nighttime searches and daytime flag collection using an IR thermometer (Kizen LLC, Walpoke, MA, USA). Temperature was recorded in two transects perpendicular to the road edge. Four recordings were made while walking each transect: at the road edge, as well as 7.5 m, 15 m, and 30 m from the road (Figure 2, transects 1 and 5). A total of 53 recording events occurred between 24 May 2021 and 8 August 2021. Temperature monitoring occurred during nighttime field observations and daytime flag collection resulting in paired sampling negating structured time periods. Carbon dioxide measurements were conducted at the same time as temperature recordings.

Data analysis. Three data points were recorded for each flag position. The three measurements included the distance of the flag to the pole (Figure 3 line D) as well as where the continuation of the tape measure crossed the tape measure along the road (Figure 3 point 1). Knowing the minimum distance from the static pole to the tape measure along the road (Figure 3 point 2), locations could be converted to coordinates on a Cartesian plane. This was accomplished by calculating the angle θ formed by measuring the shortest distance between the pole and the road edge (Figure 3 line B) the hypotenuse (Figure 3 line C), and the distance along the road between points 1 and 2 (Figure 3 line A). The angle θ was calculated using θ=cos−1C2+B2−A22CB, as labeled in Figure 3. X and Y components of the Cartesian coordinates were calculated by taking line D and multiplying sin(θ) and cos(θ) for the x and y coordinates, respectively. Calculating the distance from the release site was accomplished by finding the distance between the coordinates of the release site and the tick using the formula $Distance=\sqrt{{{(x}_{2}-x_{1})}^{2}+{{(y}_{2}-y_{1})}^{2}}$. Calculating the shortest distance between the tick and the road edge was accomplished by determining the line equation of the straight tape measured line along the road. The distance between the road line and the location of the tick was determined by finding the shortest perpendicular line from the road line to the tick.

Distances from release points and distances from the road were compared using mixed-effects models in R 4.0.0. (Released on 24 April 2020) (R Core Team 2022). The goal of the models was to determine if the proximity to the road edge was significantly different between treatment groups. Shapiro–Wilk tests were conducted on the tick distances to determine the normality of the data, which found a left skew present in the distribution of distance from the road edge and a right skew present in the distribution of distance from the release site. The square of distance from the road and the natural log of the distance from the release site were used to correct for the skews in the original data, as this showed an improvement in the *p*-values generated by the Shapiro–Wilk tests. However, the data was not transformed to preserve the quantitative meanings of the coefficients generated by the model. Shapiro–Wilk tests were conducted using the Shapiro test function in base R. Data was manipulated using the dplyr package, v. 1.1.2 [17].

Tick distances from the release point and tick distances from the road edge at sites 1 and 2 were compared using a mixed-effects model using the R package lmerTest version 3.1-3 (Released 23 October 2020) [18]. Position data of the first 40 days post release at site 1 and of the first 34 days post release at site 2 were used, as the number of ticks observed at each site visit became too small for the model beyond this cutoff.

Models employed by this study were generated using the lmer function from the package lmerTest [18], which creates a fit linear mixed-effect model that compares treatments to a control or baseline group. The movement of the no Haller’s organ and no eyes treatment groups were compared to the unmodified (control) group movements. The model was used to determine differences of tick movement with treatment and site as a fixed effect and time as a random effect.

Surface temperatures were compared using a mixed-effects model using the R package lmerTest (version 3.1-3, released 23 October 2020) [18]. Surface temperatures collected at both sites were included in this analysis. The model compared surface temperature with sample location, time, and the interaction of sample location and time as fixed effects and site as a random effect.

## 3. Results

Summary Data. Fifty control ticks, 50 no HO ticks, and 50 no eye ticks were included in the study at site 1; and 50 control ticks, 48 no HO ticks, and 50 no eye ticks were included in the study at site 2. At site 1, there were 47 observations of ticks further away from the road edge than the release point within the first 18 days of the study. Of the ticks that were found further from the road than the release point (7.5 m), 15% (*n* = 7) were in the control group, 28% (*n* = 13) had no HO, and 57% (*n* = 27) had no eyes. These observations represented 11.1% of the 420 observations, and no ticks were observed behind the release point after the 18th day at site 1. At site 2, there were 61 observations further from the road edge than the release point throughout the study. Of the ticks that were found further away from the road than the release point (7.5 m) at site 2, 23% (*n* = 14) were from the control group, 51% (*n* = 31) had no HO, and 26% (*n* = 16) had no eyes, representing 15.9% of the 384 observations. At site 2, ticks were observed further from the road than the release point until the 54th day of the study.

**Figure 4:**
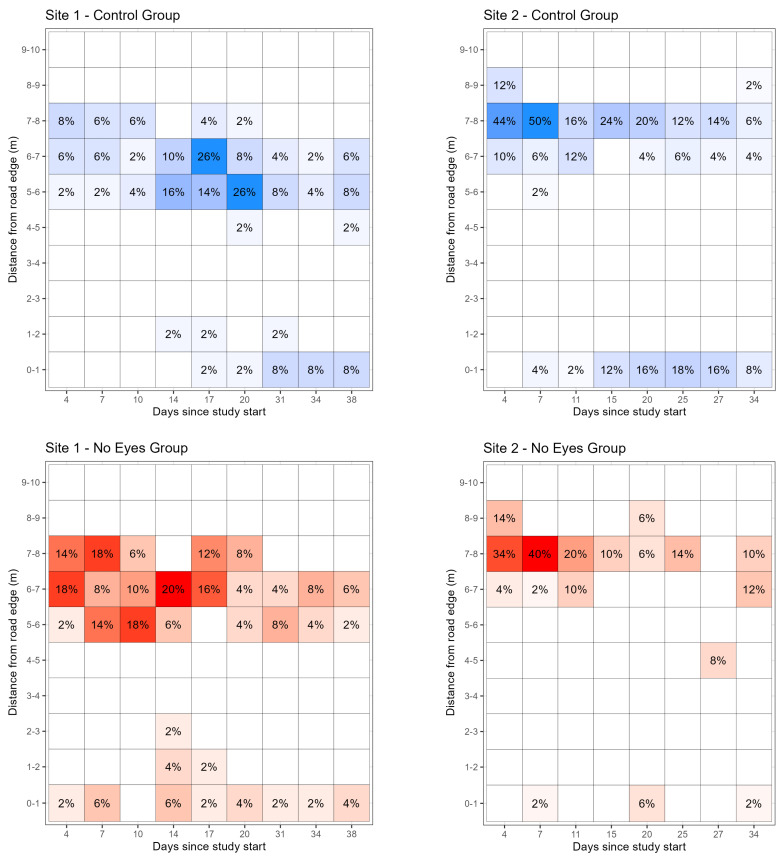

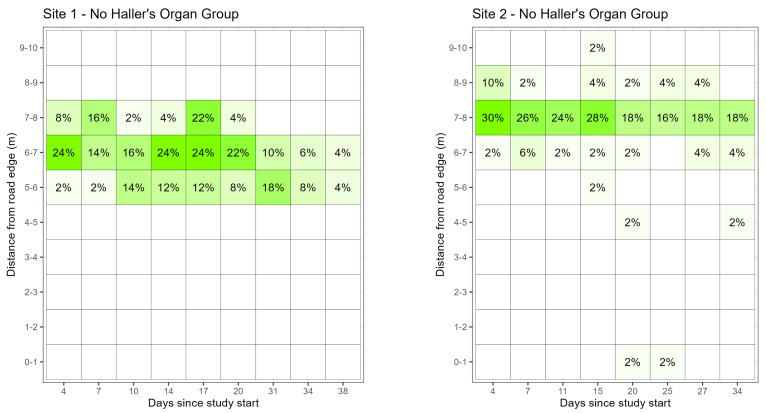
Heatmaps represent the location of released ticks over time, with color intensity representing the number of ticks found as a percentage of total ticks released (*n* = 50). The x-axis represents the number of days since tick release. This figure was produced using the R package ggplot2 [19].

Of ticks that were observed within 1 m of the road edge at site 1, 51% (*n* = 19) were in the control group, and 49% (*n* = 18) were in the no eye group, representing 8.8% of the 420 observations completed at site 1. There were no ticks from the no HO group observed within 1 m of the road edge at site 1. The first tick to be found within 1 m of the road edge at site 1 was on the 17th and 4th day of the study for the control and no eye groups, respectively. Of the ticks observed within 1 m of the road at site 2, 79% (*n* = 38) were in the control group, 10% (*n* = 5) had no HO, and 10% (*n* = 5) had no eyes, representing 12.5% of the 384 observations. The first tick to be found within 1 m of the road edge at site 2 was on the 8th, the 20th, and the 8th day of the study for the control, no HO, and no eye groups, respectively (Figure 4).

Heat and carbon dioxide stimuli were investigated at roads compared to the surrounding environment. While data are not presented in this paper, no differences were observed in CO_2_ concentration between the road surface, 7.5 m, 15 m, or 30 m from the road. Temperatures were always higher on the road surface than at any measured distance from the road during each of the 53 recording events and temperatures at all three distances were significantly lower than the road surface.

**Figure 5:**
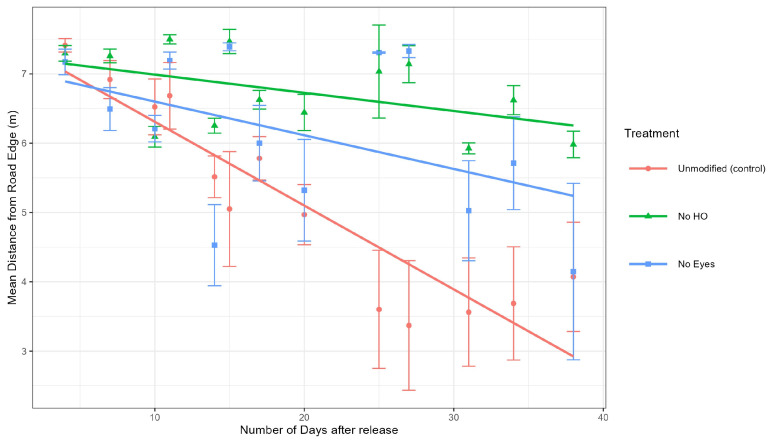
Line plot of the means of distances from road edge for sites 1 and 2. Time was determined by placement of flags after the ticks were released at the study site. Error bars are ±1 standard error. Graphs were produced using the R package ggplot2 [19].

**Figure 6:**
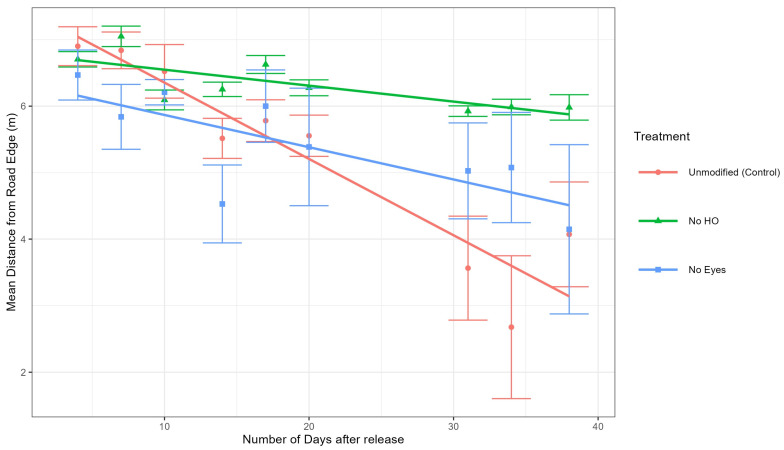
Line plot of the means of distances from road edge for site 1. Time was calculated from the number of days after the ticks were released at the study site. Error bars are ±1 standard error. Graphs were produced using the R package ggplot2 [19].

**Figure 7:**
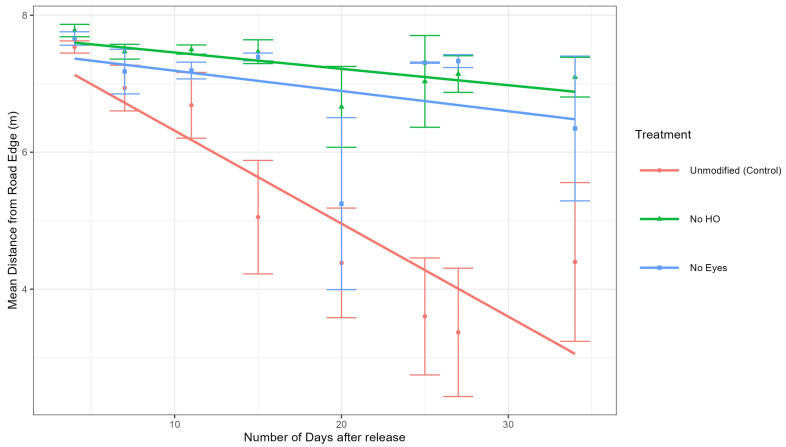
Line plot of the means of distances from road edge for site 2. Time was calculated from the number of days after the ticks were released at the study site. Error bars are ±1 standard error. Graphs were produced using the R package ggplot2 [19].

Analysis of tick positions at sites 1 and 2. To determine if the different groups (control, no HO, and no eyes) moved differently (away or towards the road), a mixed-effects model was used to calculate the differences in both distance to the road of the control group and each treatment group (no HO and no eyes groups) at sites 1 and 2 (Figure 5). The mixed-effects model also included days since tick release as a random effect to account for tick movement over time. Our model demonstrated that the no HO group was significantly further from the road edge than the control group at site 1 (estimate = 0.92 m, *df* = 740, *t* = 3.755, *p* = 0.0002) and site 2 (estimate = 0.86 m, *df* = 738, *t* = 2.548, *p* = 0.0110). The model also demonstrated that the no eyes group was not significantly different from the unmodified group at site 1 (estimate = 0.01 m, *df* = 741, *t* = 0.058, *p* = 0.9539) but were significantly different from the unmodified group at stie 2 (estimate = 1.24 m, *df* = 740, *t* = 3.462, *p* = 0.0006). Combined data is represented in Figure 5. In summary, the no HO group was significantly different from the unmodified group at sites 1 and 2, while the no eye group was significantly different from the unmodified group at site 2 but not site 1 (Figure 6 and Figure 7).

**Figure 8:**
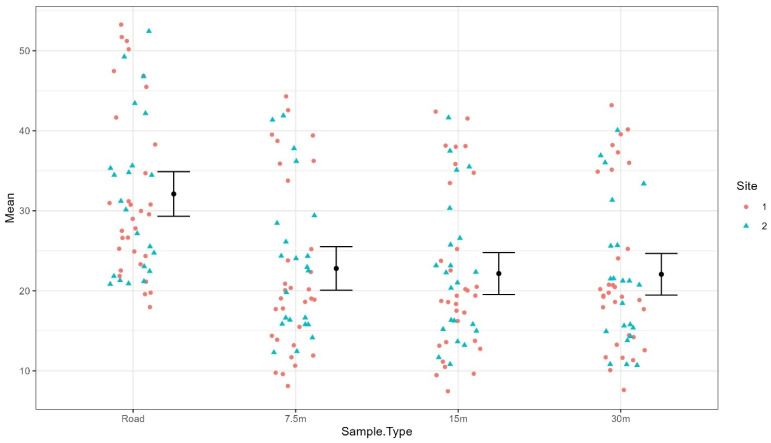
Strip chart of temperature readings at the road surfaces at 7.5 m, 15 m, and 30 m from the road surface within the field habitats (sites 1 and 2). Error bars represent 95% confidence interval. Graphs were produced using the R package ggplot2 [19].

Temperature Measurements. The mixed-effects model was used to calculate the difference between the surface temperature at 0 m, 7.5 m, 15 m, and 30 m from the road edge for both sites. Results of the mixed-effects model demonstrated that field surface temperatures at 7.5 m (mean = −9.94 °C, *df* = 204, *t* = −2.305, *p* = 0.0222), 15 m (mean = −10.05 °C, *df* = 204, *t* = −2.332, *p* = 0.0206), and 30 m (mean = −9.96 °C, *df* = 204, *t* = −2.312, *p* = 0.0218) from the road were significantly lower compared to road temperatures (Figure 8). Mean road surface temperatures were an average of 9.8–10 °C hotter than field surface temperatures at 7.5 m, 15 m, and 30 m. Despite a wide range of surface temperatures (14.6–53.3 °C for road surfaces, 7.5–45.0 °C for field surfaces), roads were always warmer than the surrounding field surfaces at all distances.

## 4. Discussion

Data from this experiment supported the hypothesis that adult unmodified *D. variabilis*, with intact HO, move towards and congregate along road edge habitat. The mixed-effects model utilizing data from sites 1 and 2, demonstrated that the unmodified group moved significantly closer to the road edge over time than the no HO treatment group at both sites and the no eyes treatment group at site 2. Ticks in the no eyes treatment group were not significantly different from the unmodified group at site 1 (Figure 6) but were more similar to the no HO group at site 2 (Figure 7). Modified ticks did not consistently act as expected, especially at site 2. Our model was designed to be conducted with site as a random effect; however, the limited number of study sites made it very undesirable to calculate variance requiring site to be included in the model as a fixed effect. It is possible that the model would have supported our hypothesis, including attraction of the no eye group with intact HO to road edge habitat, if more site replications were included in our model. Out data suggests that the HO plays a significant role in attraction of adult *D. variabilis* to road edge habitat, but we are unable to eliminate the role of eyes in attraction due to variations between study sites and lack of replication limiting our model.

Higher road temperatures existed when compared with surrounding fields during all 53 measurement periods in this study, irrespective of the time of day. The temperature difference between the road surface and the ground in the surrounding field was greatest in the first hours after sunset (between sunset and 12 a.m.). During this time, the road continued to retain heat from the day while the field surfaces cooled more rapidly. This was likely due to the high specific heat of traditional asphalt roads [20], which allowed the road to retain heat to a greater degree. Since adult *D. variabilis* are attracted to heat [4] and roads are consistently hotter than their surrounding environment, the latent heat absorbed by the road may be acting as a beacon for movement during cooler evening hours. This drastic temperature difference during the evening may be critical for tick migration towards road edge habitat during questing activity. No differences in CO_2_ concentrations were detected between the road or distances sampled away from the road; however, CO_2_ should continue to be investigated as a potential attractant toward road as dry ice has been an effective attractant for *D. variabilis* adults [21].

Questing occurs more frequently in adult *D. variabilis* with increased solar radiation levels and longer photoperiods [22,23] primarily during daylight hours. While not questing, ticks need to rehydrate [24] and are likely to descend vegetation to enter microclimates with higher humidities. If a stimulus is encountered while on the substrate, *D. variabilis* adults will move towards that stimulus [4]. The migration of *D. variabilis* adults to the road edge habitat observed in this study is likely best explained by temperature differences between the road surface and surrounding substrate, resulting in adults moving toward that stimulus during the cooler nighttime hours when the temperature differential is greatest. Random walking [25] is unlikely to account for 45% (5/11) at site 1 and 50% (9/18) at site 2 of recovered ticks on days 31 and 25, respectively, of the study moving a minimum of 2 m from the road edge (Figure 4). While behavioral patterns differ between tick species, a study by Romanenko et al. [16] described use of the random walk formula. They compared previously published results for *Ixodes persulcatus* to their current study and calculated, factoring time questing and time rehydrating, that in a 20-day period, *I. persulcatus* likely randomly moved in a radius no greater than 61.8 cm from its origin without a stimulus. It is highly unlikely that nearly half of detected *D. variabilis* adults at our study sites around 30 days from release would move at least 5.5 m without a stimulus.

Our observation concerning the attraction of adult *D. variabilis* towards road edges is consistent with the findings of McEnroe [12] and Stewart [13], who both observed the congregation of marked *D. variabilis* along road edge habitat. Additionally, our study suggests that the HO plays a critical role in the attraction of adult *D. variabilis* towards roads as ticks in the control group (with intact HO) were significantly closer to the road than those with the HO removed. Additional experiments are needed to better understand the function of eyes and their role in attraction to roads. At both study sites, the no HO groups appear to drift towards the road without migrating to road edge habitat with the same intensity as the unmodified ticks (Figure 6 and Figure 7). This could be due to dwindling tick observations in the later days of the study or could represent a mild sampling bias resulting from closer transect spacing in front of the release point (Figure 2).

Nymphs and larvae of several species of ticks were potentially present in the study sites during the timeframe of our study [26,27]; however, none were found at the road edges or along transects sampled between May 24 and August 8. These life stages likely would have been found via dragging but were outside the scope of our study. Like the differences found between immature stages and adults of *A. americanum* by Briggs et al. [28] for CO_2_ attraction, immature *D. variabilis* may not be attracted to road stimuli like adults (even though *D. variabilis* possess HO at this life stage) [29]. The possibility also exists that the immature life stages are unable to move significant distances towards stimuli because of their small size. While no reports were found in the literature of other tick species or life stages demonstrating an attraction to roads (like many species are attracted to CO_2_) [30,31,32], no other heterospecific ticks in any stage were encountered in this habitat during this study. In previous unpublished studies at this site, immature *D. variabilis* had only been collected from small mammals trapped at State Game Lands 169 [33]. Larval, nymphal, and adult *I. scapularis* have been collected through dragging for other studies in nearby forested habitat but were not observed during this study [33]. Heat is a source of attraction for adult and nymphal *I. scapularis* and *A. americanum* at short distances up to 5 cm [34], but additional studies need to be conducted to determine if these attractions towards heat are biologically relevant at a landscape level.

Roadside ecology and the epidemiology of tick-borne diseases is an understudied facet of surveillance [35], despite an abundance of these regions in tick habitats (which often correspond with human activity). Our study demonstrated that unmodified adult *D. variabilis* are attracted from at least 7.5 m to asphalt roads, possibly by using the HO to detect heat; this does not preclude the possibility for attraction from greater distances, which warrants further study. Conclusions regarding tick affinity for roadside habitats which coincide with human activity may be useful as a tool for risk assessment and alternative management techniques regarding control of these vector populations. Our observations suggest an increased probability of humans as accidental hosts by questing ticks along asphalt road edges. In addition, adult *D. variabilis* were routinely observed questing on long blades of grass hanging directly over the road edge during daytime flag collections, suggestive of an enhanced risk to encounter ticks for people traversing the roads at this study site (the State Game Lands is commonly used for biking, fishing, hiking, etc.). While it is well established that *D. variabilis* is a vector for *Rickettsia rickettsii* (causative agent of Rocky Mountain Spotted Fever) and *Francisella tularensis* (causative agent of tularemia), other pathogens (e.g., Powassan virus), are continually being evaluated in this tick species [36,37]. As this species continues to expand its range throughout North America [38], research aimed at better understanding habitat preferences, host-seeking stimuli, and seasonal movement patterns will be necessary to fully understand their potential as a public health nuisance.

The congregation of *D. variabilis* along road edges also denotes a probable control strategy for this species throughout their range. Road edges could potentially represent a “trap crop” location. Traditionally, a trap crop is an alternate crop that is planted near a desired crop and is at least as desirable for an insect group as the desired crop to act as a decoy/trap for herbivores. This decreases damage to the desired crop and creates a nearby area to control insects, without directly applying insecticides to the desired crop. Road surface temperature functions in a similar manner as it attracts *D. variabilis* to road edge habitat, primarily because of the high heat capacity of the road compared to the surrounding environment. Ticks “trapped” in road edge habitat can be controlled through acaricide application or potentially mowing the grass to very short lengths during the period when they are most abundantly found along roads. While conducting a single mowing event along recreational hiking trails does not appear to reduce *D. variabilis* abundance [39], creating an inhospitable microhabitat without much moisture along road edges that attract *D. variabilis* adults may reduce human contact with the vector. Consequently, the results of this study present novel opportunities for the development of previously unutilized methodologies that could be instrumental in the control of *D. variabilis* populations.

Our findings suggest that surfaces with increased temperatures (e.g., paved recreation trails, basketball courts, park pavilions, etc.) may also possess increased adult *D. variabilis* loads along their edges. The knowledge regarding congregation of this tick species along road edges should be considered by land managers (particularly in especially busy/highly traversed recreational areas) to strategically reduce tick populations through localized acaricide treatments or mechanical culling. These findings also reinforce the need for tick safety during any outdoor activities, as high adult *D. variabilis* tick loads along paved surfaces results in an increased possibility of humans/domestic animals becoming accidental hosts. Moreover, no ticks were assessed during this study with both painted eyes and HO removed. We hypothesize that ticks without access to both sensory organs would display limited and/or a lack of movement towards the road edge. Future studies should consider including an experimental group of ticks with both sensory organs covered or removed. Additionally, investigation of various tick species (with consideration of seasonality and abundance at the selected field site) should be conducted to better define the role of the eyes and HO across tick species.

## Data Availability

The original contributions presented in the study are included in the article; further inquiries can be directed to the corresponding author.
